# Categorization of everyday sounds by cochlear implanted children

**DOI:** 10.1038/s41598-019-39991-9

**Published:** 2019-03-05

**Authors:** Aurore Berland, Edward Collett, Pascal Gaillard, Michèle Guidetti, Kuzma Strelnikov, Nadine Cochard, Pascal Barone, Olivier Deguine

**Affiliations:** 1 UMR 5549, Faculté de Médecine Purpan, Centre National de la Recherche Scientifique, Toulouse, France; 2 Centre de Recherche Cerveau et Cognition, Université de Toulouse, Université Paul Sabatier, Toulouse, France; 3 Unité de Recherche Interdisciplinaire Octogone, EA4156, Laboratoire Cognition, Communication et Développement, Université de Toulouse Jean‐Jaurès, Toulouse, France; 4 Faculté de Médecine de Purpan, Toulouse, France; Service d’Oto‐Rhino‐Laryngologie et Oto‐Neurologie, Hopital Purpan, Toulouse, France

## Abstract

Auditory categorization is an important process in the perception and understanding of everyday sounds. The use of cochlear implants (CIs) may affect auditory categorization and result in poor abilities. The current study was designed to compare how children with normal hearing (NH) and children with CIs categorize a set of everyday sounds. We tested 24 NH children and 24 children with CI on a free-sorting task of 18 everyday sounds corresponding to four a priori categories: nonlinguistic human vocalizations, environmental sounds, musical sounds, and animal vocalizations. Multiple correspondence analysis revealed considerable variation within both groups of child listeners, although the human vocalizations and musical sounds were similarly categorized. In contrast to NH children, children with CIs categorized some sounds according to their acoustic content rather than their associated semantic information. These results show that despite identification deficits, children with CIs are able to categorize environmental and vocal sounds in a similar way to NH children, and are able to use categorization as an adaptive process when dealing with everyday sounds.

## Introduction

When an infant is identified as having profound-to-total bilateral hearing loss, the surgical implantation of cochlear implants (CIs) is envisaged as a means of restoring his or her access to auditory information. The benefits of CIs for speech perception in children with prelingual hearing loss have been widely documented over the past three decades, resulting in an extension of prescription criteria^1,2^. One of the greatest proven benefits of CIs is speech comprehension^3,4^. Alongside improvements in oral language skills, speech comprehension is seen as one of the key objectives of rehabilitation with CIs.

A CI does not function in the same manner as a conventional hearing aid (i.e., by contributing to the amplification of sounds). Instead, it turns sounds into trains of electrical pulses that directly stimulate the auditory nerve. This information is then relayed to the auditory centres of the brain, which in turn transform the impulses into auditory percepts. However, the auditory information provided by the implant is spectrally degraded^5^ and lacks the fine spectrotemporal information that is crucial to certain aspects of speech comprehension - most notably the perception of prosodic information^6–9^. This means that other aspects of auditory perception are also problematic for CI users, such as the identification and recognition of environmental and musical sounds.

## Development of nonlinguistic sound perception

The recovery and/or development of speech comprehension is normally seen as the main goal of CI implantation. Accordingly, only limited attention has been paid to the perception of nonlinguistic sounds in CI users.

For example, whilst CIs may enhance users’ awareness of their immediate auditory environment^10^, a substantial deficit remains in the perception and identification of specific environmental sounds^11–13^, that is, those not classified as speech or music. This deficit has a negative impact on how CI users interact with their environment, and it is therefore important for more studies to be conducted on this topic.

To our knowledge, the identification of nonlinguistic sounds by implanted adults with postlingual deafness has only been reported in a small number of studies^11–15^. There have been even fewer studies among children with CIs^16,17^. Furthermore, owing to a lack of standardized protocols for assessing auditory performance and the use of different evaluation procedures (open-set or closed-set identification assessments, written words or images, different sound stimuli, in different conditions) it is difficult to identify precisely why CI users struggle to identify nonlinguistic sounds.

Studies among implanted adults have found the mean recognition accuracy of nonlinguistic sounds to be between 70% and 80%, when assessed using a closed-set (i.e., participants know which stimuli to expect) recognition test^12,13^. This performance falls drastically to 30-40% when an open-set response format is used^11,12,15^.

Studies in children with CIs have also reported poor performances on environmental sound recognition^16,17^. Liu *et al*.^16^ administered the Sound Effect Recognition Test^18^ to a large cohort of children with prelingual deafness. With a closed-set format, mean identification accuracy across all participants was 68%. Quaranta *et al*.^17^ obtained similar results, with a mean identification accuracy of 59% for common environmental sounds in children with CIs.

These results show that it is crucial to improve current understanding of the factors involved in the recognition and identification of nonlinguistic sounds in implanted children. A better grasp of these mechanisms could provide important information for developing rehabilitation tools and coding strategies with which to enhance CI users’ performances. Moreover, improving children’s performances could lead to better development in adult life.

Studies of CI users, both adults and children, have classically highlighted two groups of factors favouring auditory perception abilities. First, those related to the individuals and their deafness, which include age, duration of deafness, aetiology, and experience with their CIs. Second, those concerning the acoustic characteristics of the sounds and the extent to which CIs can accurately process and represent these characteristics. Regarding the latter, each sound can be described by its spectral (modulations, centroid, etc.) and temporal (onset, amplitude modulations, duration) structure, and identified accordingly. For example, CI users have difficulty recognizing complex sounds with rapid changes in temporal and spectral content^13,19^. By contrast, harmonic or repeated sounds with simple temporal or spectral content are more easily identified^12,20,21^.

In addition, the wide variety of nonlinguistic/environmental sounds produced by a variety of different sources makes it difficult to study the acoustic characteristics that are important for environmental sound perception.

## Auditory categorization

Perception of everyday sounds more often than not takes place in environments where many sounds occur simultaneously and cannot, therefore, all be attended to at the same time. Part of dealing with these situations involves the categorization of sounds. The characteristics of a sound source can be quickly inferred from pre-existing category knowledge, as has been shown for the categorization of phonemes and voice gender, as well as the materials and actions that cause different sounds^22,23^. According to Gaver^24^, auditory categorization involves two different strategies. The first, referred to as *everyday listening*, involves extracting semantic information about the sound source, specifically the object and/or action that created the sound (e.g., identifying a sound as wooden or metallic, produced by the action of hitting or rubbing). This strategy also applies to humans and other living beings and their actions. The second strategy, *musical listening*, involves the perception of qualitative aspects of the sound, (e.g., pitch, loudness, timbre) and also its emotional content (pleasant or unpleasant). Auditory categories have been found to be based predominantly on information extracted via *everyday listening*, and broadly include human and animal vocalizations, impact sounds, water sounds^25^, and living and nonliving sounds^26^.

## Aim of the study

In order to further understand the ability of children with CIs to perceive everyday sounds, we adopted a free sorting task (FST) protocol^27,28^. There are several advantages to using the FST to test children with CIs, as it allows a set of natural stimuli to be studied in a single test without imposing specific performance levels. Any and all categorization strategies are possible, participants may create as many or as few categories as they choose, and the task can be easily performed by 6- to 9-year-old children (see Berland *et al*.^28^, for normal-hearing children).

This task requires participants to group auditory stimuli into as many groups as they wish. They may do so using any categorization strategy they choose, basing their groupings (or categories) on the sounds’ shared semantic and/or acoustic properties^12^. This process is closely related to that of similarity judgments, involving either holistic-based decisions^29^ or analytical processes, depending on the context^30^, and is also strongly influenced by cognitive factors^25,31^.

Importantly, participants are not required to identify the stimuli, so categories may be created with or without correct identification. Uncovering the shared properties of categories, even when sounds are not identified, could shed light on how children with CIs perceive everyday sounds in a more real-world scenario. For example, using an FST makes it possible to see which categories - and therefore which kinds of sounds - are most salient for children with CIs. This differs from standard categorization tasks, where the question posed to participants is often “To which of the given categories does the following stimulus belong?”, rather than (in the current case) “What are the categories into which you can divide these stimuli?”

We also performed further analysis of the FST results, in order to uncover possible links between the participants’ categorization strategies and the acoustic properties of the stimuli, as well as the aetiological data of the implanted children, with a view to understanding how the performance of the individual child and the processing capability of the CI affect the perception and categorization of sounds.

The present study also compared implanted children with their normal hearing (NH) peers. Results show that, when categorizing a set of natural everyday sounds (including human voice, musical sounds, and environmental sounds), both groups tend to adopt similar listening strategies and define comparable semantic categories. However, as also demonstrated in implanted adults^27^, children with CIs tend to develop categories that are more strongly based on acoustic characteristics, probably owing to a deficit in their ability to identify sounds and extract semantic information.

## Materials and Methods

### Participants

We tested two groups of participants: 24 children who are prelingually deaf with unilateral implants (*M*_ag*e*_ = 9.24 years, *SD*_age_ = 1.21), and 24 NH children with a similar age distribution (*M*_age_ = 8.81 years, *SD*_age_ = 0.86), as shown in Table 1. We initially recruited 30 implanted children, but six failed to complete the task, owing to a lack of attention or difficulty performing the task. It was not possible to obtain any data from these six participants, and they were therefore excluded from the dataset.

**Table 1:** Description of the participants and individual characteristics of Normal Hearing children (NH) and children with CI (ages and age at evaluation in months).

Group	n	Age (years)			Female/Male	
		Mean ± sd	Min.	Max.		
CI children	24	9.2 ± 1.2	6;2	11;2	9/15	
NH children	24	8.8 ± 0.9	6;1	9;11	13/11	
**ID**	**Gender**	**Age at assessment (months)**	**Age at implantation (months)**	**Experience with CI (months)**	**Disyllabic word (%)**	**Sentence open list (%)**
CL	M	93	18	60	100	100
DK	M	74	31	44	70	97
JL	M	103	33	68	80	52
AC	F	85	22	48	100	92
LM	F	95	14	79	95	100
MS	F	110	24	85	100	100
BT	M	109	30	78	100	100
ST	M	108	42	64	100	72
AM	F	108	23	83	95	100
MR	F	114	49	63	100	100
GP	M	124	30	92	80	90
JA	M	116	28	86	90	100
RN	F	113	19	93	100	100
RK	M	107	24	80	85	72
CA	M	113	33	78	100	77
DV	M	97	14	82	100	100
DB	M	116	32	83	85	88
JA.2	M	121	30	89	100	100
LM.2	F	113	20	91	100	100
BT.2	M	122	32	89	90	83
MP	M	128	14	64	100	97
PA	M	133	18	55	90	94
CA	F	125	44	32	80	100
LJ	F	134	19	52	48	20

The audiological evaluation (word and sentence) is expressed in % correct.

None of the 30 children with CIs presented any disorders associated with deafness, and all were monitored by the paediatric cochlear implant programme in Toulouse. All 24 successfully tested children had been fitted with a conventional 22 electrodes CI, except for one who had been implanted with a CI 24 electrode array. Their mean chronological age at implantation was 2 yr 2 mo (range = 1 yr 2 mo - 4 yr 1 mo, *SD*_age_ = 0 yr 9 mo) and their mean hearing experience with their CI was 6 yr 4 mo (range = 2 yr 8 mo - 7 yr 7 mo, *SD*_age_ = 1 yr 5 mo).

The NH children (control group) were recruited in primary schools or recreational centres, and were part of a larger cohort described in the previous study^28^. Each child had normal hearing and normal or corrected-to-normal vision, and no auditory, neurological or psychiatric disorders were reported. Both groups of children also had similar levels of education for their age.

This study was approved by the Sud-Ouest et Outre-Mer I institutional review board (no. 11 227 02) and the experiment was conducted in accordance with the Declaration of Helsinki (2013). Prior to their inclusion in the study, all participants and their parents (or legal guardians) gave their full written informed consent.

### Materials

All participants were assessed with an auditory FST presented via the open-source software TCL-LabX^32^, as described in previous studies^27,28^. We selected 18 sounds from a larger set of 45 taken from a web database (http://www.freesound.org/). They were chosen to cover a broad range of everyday sounds (Table 2) that corresponded to four main a priori categories: nonlinguistic human vocalizations, environmental sounds, musical instruments, and animal vocalisations. Having a different number of stimuli in a priori categories enhanced the objectivity of the categorization, as it avoided the natural tendency to create groups containing equal numbers of items (i.e., the arithmetic strategy of dividing the stimuli into two groups, then each of these into two subgroups is discouraged by the unpredictable number of stimuli in each one). The categories we selected were easily identified by a cohort of 15 NH French adults, and were considered to be prototypical for their category (see Berland *et al*.^28^. All stimuli were monophonic and recorded in.wav format with a sampling frequency of 44,100 Hz. They were normalized in both duration (2 s) and loudness, using overall root-mean-square (RMS) power. The acoustic properties of all the sounds are provided in Supplementary Table 1.

**Table 2:** Stimuli included in the Free sorting Task and grouped in *a priori* classes.

Category	Sub category	Description	Stimuli ID
Human non-linguistic vocalizations	Child	Babbling	bab
		Crying	cry
	Woman	Cough	cgh-f
		Laugh	lgh
	Man	Yawn	yawn
		Scraping throat & cough	cgh-m
Animal vocalizations	Animals	Bird	bird
		Cow	cow
Environmental sounds	Alert	Horn	horn
		Bell	bell
	Everyday sounds	Front door	dr
		Rustling of paper	ppr
Musical instruments	Stringed instruments	Violin	vln
		Double bass	bas
	Wind instruments	Flute	flute
		Tuba	tuba
	Percussions	Kettledrum	k-drm
		Drum kit	drm

### Procedure

We implemented a procedure similar to that used by Berland *et al*.^28^. Each participant sat in a quiet room, in front of a PC monitor positioned at eye level, with the experimenter sitting next to him or her. For the NH children, the stimuli were presented in stereo via Sennheizer headphones. For the implanted children, the stimuli were presented in free-field conditions, via two Creative loudspeakers each positioned 40 cm from the participant. In both cases, the intensity/volume was adjusted to 65 dB SPL. The hearing deficit of the non-implanted ear was 87–120 dB in our patients so that residual hearing could not influence the results at this level of sound presentation.

Using TCL-LabX software, the 18 sounds were represented on the computer screen by 18 numbered and coloured squares that were initially identically arranged for all participants. The task consisted in listening to the 18 sounds and placing them in groups (i.e., creating categories using any means and criteria). Participants double-clicked on each square with the computer mouse to hear the corresponding sound, and categories were created by dragging and positioning squares together on the screen.

Participants were instructed to “Group the sounds that go well together. You can make families of one, two, three, or more sounds, as you wish”. No further instructions were given concerning the categorization criteria. Minimal feedback was given by the experimenter, in order to facilitate the completion of the experiment. Sign language was used with oral instructions when necessary, and the instructions were reworded if needed. When children’s attention strayed, the experimenter re-focused them on the task.

No time limit was given for the experiment, and the sounds could be listened to multiple times and in any order. Once participants had decided that their categorization was complete, they were not allowed to modify it. After the FST, participants were asked to define and name both the categories they had created and the individual sounds. This last step was done in order to verify/assess their ability to identify everyday/natural sounds. No such naming was asked of the NH children in the original study^28^, as the ages of the participants corresponded to developmental stages where sounds are not yet recognized, except for very typical ones.

## Analyses

### Identification performance analysis

As indicated above, the implanted children were asked to name/identify the sounds in an open-set identification task after they had completed the FST.

Open-set tasks are important to estimate participants’ cognitive representations of sounds, but the results are difficult to evaluate. A broad range of responses from participants could constitute correct identification. More specifically, if participants could signify by one means or another (oral or French Sign Language) that they had identified the sound, either by naming, describing or explicitly mimicking the sound, its superordinate category, or a close sound from the same category, we considered that the sound had been correctly identified.

However, responses were also submitted to a more accurate coding strategy, which is described below and set out in Table 3.

**Table 3:** Distinction of the different response categories of the CI children during the identification task proposed after completion of the Free Sorting Task.

1	A	Correct and stable identification
	B	Fair but not stable identification
2	C	Identification with a sound close to the proposed sound
	D	Identification by the superordinate category
3	E	Confused
	F	Not identified

For each sound and each a priori category of sounds, we calculated a percentage of identification, using the following coding:

#### Correctly identified sounds

(Response category 1): correct and stable (i.e., repeated) responses were placed in Response category 1 A. If participants generated responses that were approximately correct but unstable (alternative or confused responses), these were placed in Response category 1B.

#### Partly identified sounds

(Response category 2): this included two types of identification, either a sound similar/related to the one that had been heard (Response category 2C) or a correctly identified superordinate category^33^ (Response category 2D). The a priori categories we had previously defined (cf. Table 2) were taken to be the superordinate categories for musical sounds and nonlinguistic human vocalizations. For instance, we accepted *music* for musical instruments, and *a person*, or *baby*, *child*, *dad*, *mom*, *man*, or *woman* for nonlinguistic sounds, regardless of the accuracy of the information. For all other environmental sounds, any circumlocution describing the category was accepted. Regarding the notion of *proximity* (also referred to as *typicality*^33^), all human vocalization sounds were deemed to be close for nonlinguistic human vocalizations, as were sounds from the same family of musical instruments (brass, woodwind, strings, percussion) for musical sounds, sounds from the same animal’s family (bird, mammal, etc.) for animal vocalizations, and sounds from the same type of source (alarm, etc.) for environmental sounds.

#### Not identified or poorly identified sounds

(Response category 3): sounds were considered to be nonidentified if no response was provided, or if the answer was assumed to have been randomly assigned. A sound was considered to be poorly identified when a child incorrectly placed it in the same a priori category as a sound that could not be considered to be *close*, (Response category 3E) or identified it incorrectly (Response category 3F), naming a sound that belonged to a different a priori category.

### Acoustic analysis

We also analysed a range of acoustic characteristics, based on a previous study in implanted adults^27^. Five different acoustic domains were explored:

- pitch measures (mean and median fundamental frequency (F0), standard deviation of F0, maximum F0, mean fundamental pitch salience, maximum fundamental pitch salience;
- spectral measures (centroid, skew, kurtosis, mean centroid, centroid velocity, centroid uniformity and centroid standard deviation);
- temporal-envelope statistics (number of peaks, mean peak, number of bursts, mean burst, total burst duration, duration ratio computed from the broadband temporal envelope of each sound);
- periodicity measures (number of peaks in the envelope autocorrelation, maximum autocorrelation peak, mean and standard deviation of autocorrelation peaks, range of data), cross-channel correlation;
- long-term root-mean-square (RMS) measures (without weighting).

F0, an acoustic parameter, is a correlate of periodicity pitch, a perceptual parameter, and for brevity’s sake we sometimes refer to these features as *frequency*, *max frequency*, and so on. Detailed descriptions of these acoustical properties are provided in Collett *et al*.^27^ and acoustic properties of each presented sound are provided in Supplementary Table 1. The acoustic analyses were identical to Gygi *et al*.^25^ using their MATLAB scripts.

### Statistical analysis of FST data

#### Category Analysis

To analyse the categories created by the participants, we used two different functions in R Core Team software. First, multiple correspondence analysis (MCA) was applied to an indicator matrix yielded by the TCL-LabX software. MCA is an extension of correspondence analysis (CA) that allows data containing more than two categorical variables to be summarized and visualized. It can also be regarded as a generalization of principal component analysis (PCA), when the variables to be analysed are categorical instead of quantitative. The MCA used CA to represent each sound as a data point in an *n*-dimensional Euclidean space, based on categorical values (i.e., categories created by the participants). Each dimension was chosen to account for the largest amount of variance possible within the dataset, and dimensions were listed in descending order of explained variance. The MCA also performed an analysis on participants, to determine how strongly individual results coincided with the dimensions, thereby allowing us to determine the similarity of participants’ categorization strategies^34^. A total of 10 dimensions were used in the analysis, and we retaine those that explained 5% or more of the total variance. This led to the first five dimensions being used for hierarchical clustering based on principal components. We focused on the two most significant dimensions (Dimensions 1 & 2), as they accounted for the greatest variance in the data.

### Hierarchical Clustering Analysis

Based on MCA, hierarchical clustering based on principal components (HCPC) computes hierarchical clustering, and then performs *k*-means clustering. A form of partitioning clustering, *k*-means clustering is used to split a dataset into several groups. HCPC allowed us to visualize the hierarchical structure of classification in the forms of dendrograms, and thus complemented the MCA visualisation of the subjective importance of categorization features. When using this analysis, it is not possible to account for all the variance (inertia) within the data (i.e., variability of participants’ responses), meaning that some remains unaccounted for. By increasing the number of desired categories, this inertia can be reduced, and this process can be used to determine the final number of categories. If the number of categories is Q, then the optimum number of categories will be found when the change in inertia is greater when moving from Q − 1 to Q than from Q to Q + 1.

We calculated the cophenetic correlation coefficient (CCC), to measure of how accurately the distances between items in the raw data were preserved in the dendrograms. The higher the CCC values, the more representative the dendrograms of the raw classification data.

### Acoustic and history analysis

We performed statistical testing in the form of ANOVAs and *t* tests on participants’ performance data (recorded by TCL-LabX) to assess whether any significant effects of participants’ group or type of sound could explain the categorization. As well as examining correlations with acoustic information, we analysed correlations between the implanted children’s coordinates on the MCA dimensions and their audiological characteristics: age at testing, duration of deafness, duration of implantation, and audiological performance (disyllabic word recognition, sentence-in-noise recognition).

## Results

**Figure 1:**
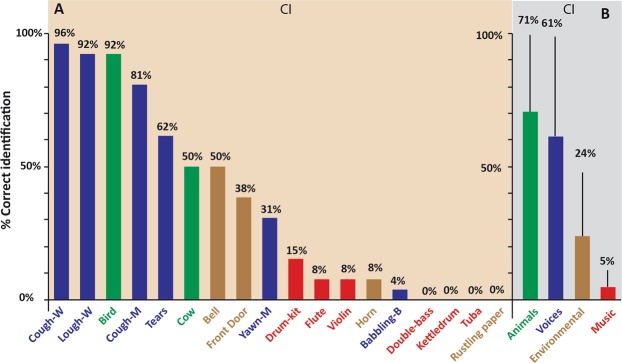
Bar chart showing percentages of correct sound identification by children with CIs. In Panel A, the 18 everyday sounds are ranked according to the percentage of correct and stable (Response category 1) identification (see Table 3). Different colours are used to indicate the four *a priori* categories of sounds: nonlinguistic human vocalizations (blue), musical sounds (red), environmental sounds (gold), and animal vocalizations (green). Panel B provides the mean identification performance (correct and stable: Response category 1) for each of the four *a priori* categories, showing that human vocalizations and animal vocalizations were both well identified by the children with CIs.

Results are provided for each of the four a priori categories of sounds^28,35^. Based on our very broad criteria (Response category 1 and Response category 2), 30.8% of environmental sounds, 41% of musical sounds, 80.8% of animal vocalizations, and 86.5% of nonlinguistic human vocalizations were correctly verbally identified. A paired *t* test highlighted significant differences in identification scores between environmental sounds and animal vocalizations (*t* test = 8.06, *df* = 25, *p < *0.001), and between environmental sounds and nonlinguistic human vocalizations (*t* test = −11.21, *df* = 25, *p < *0.001) (Fig. 1). However, the difference between environmental sounds and musical sounds was not significant (*t* test = −1.82, *df* = 25, *p* = 0.081).

We also provide data on the verbal identification of sounds by the implanted children, although this approach does have limitations when applied to children with limited verbal capacities. When a more conservative criterion was adopted (Response category 1 only), performances were much poorer (see Fig. 1), though with the same tendencies with respect to the a priori categories. We present identification scores for a priori categories and chosen criteria in Supplementary Table 3. The hierarchical clustering analysis of the whole set of stimuli was more valid, as it objectively classified responses based on their grouping, without relating them to the children’s verbal capacities.

### Hierarchical clustering: overview of categorization

**Figure 2:**
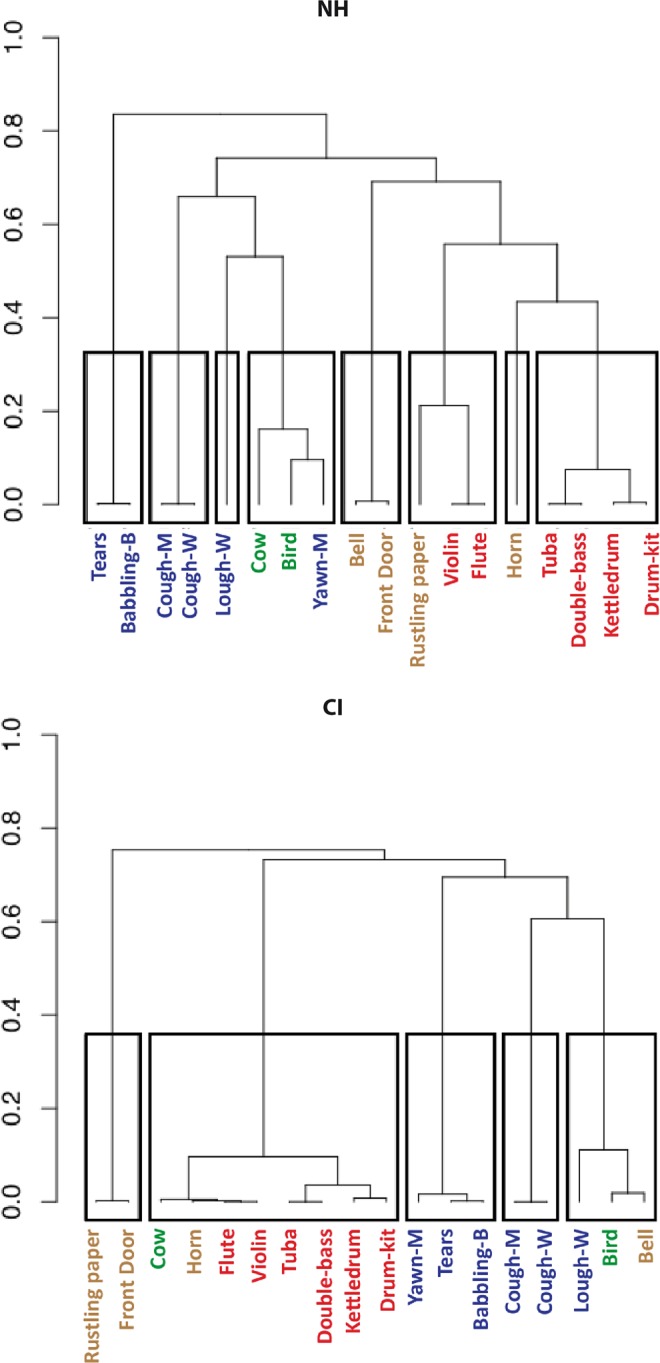
Hierarchical clustering dendrograms. The left panel shows the categorization of stimuli by NH children, and the right panel shows the categorization by CI users. The overall categories yielded by the hierarchical clustering analysis (HCPC) are indicated by rectangles, while the upper limits of these rectangles indicate the points at which each dendrogram was cut (as described in the main text). The HCPC used the dimensions selected from the MCA analysis: seven for the NH group and four for the CI group (see Table 4). The height indicates the perceptual distance between each stimulus, such that the greater the height, the more dissimilar the two stimuli were deemed to be by participants, and vice versa. Stimuli are labelled using the abbreviated sound IDs in Table 2.

The results of the FST are represented as dendrograms in Fig. 2, the direct output of the HCPC analysis. The boxes correspond to the categories selected for each group, based on the change in statistical inertia (as described in the Materials and Methods section). A total of eight categories were selected for the NH group, compared with only five for the CI group, although it should be noted that analysis of participants’ performance failed to reveal any significant difference between the groups on the number of categories created. The CCC values, which reflected how well the dendrograms preserved the original data, were 0.71 for the NH group and 0.84 for the CI group, suggesting that the NH dendrograms were slightly less representative of the participants’ strategy than the CI dendrograms.

The ordinates of dendrograms reflect the perceptual similarity between two stimuli, with a large distance indicating that the stimuli are strongly dissimilar according to the participants’ categorization. The mean intercategory distance for the NH and CI groups was found to be 0.72 for both populations, while the intra-category distance was 0.08 for NH and 0.056 for CI. However, the latter difference was found to be nonsignificant (two-tailed *t* test, *p* > 0.05).

Qualitatively, the dendrograms appeared to be different. For the NH group, many categories consisted solely of pairs of similar sounds such as the two coughing sounds (*cgh-m* and *cgh-f*), baby sounds (*cry* and *baby*) and other human vocal sounds (*yawn* and *lgh*). By contrast, the CI tree diagram was dominated by a single large category of eight mainly musical sounds. Like their NH counterparts, the implanted children also paired the coughing sounds in a separate category, and categorized the baby sounds with the sound of yawning, all three being nonlinguistic human vocalizations. The distinction of the baby sounds may have been more prevalent for NH children, as it was the first branching on the dendrograms, at 0.84 in NH children, but only the third for the implanted children, at 0.69.

Another similarity between the NH and CI groups was the grouping of musical sounds, which can be seen on the second branch of the dendrograms, at 0.76 for NH and 0.75 for CI. However, NH children could distinguish between the melodious violin/flute and the other musical sounds, which were either held notes (tuba/double bass) or percussive (kettledrum/drumkit). Children with a CI only created one category of musical sounds, and also included the sounds of a cow and a car horn. Finally for NH children, the musical sounds (specifically the violin and flute) were often accompanied by the rustling of paper, a sound which is often confused with a round of applause.

## MCA: details of categorization strategies

To assess the details of the categorization strategies employed by the two groups of participants, we applied MCA to the categorical responses. A total of 10 dimensions were used as part of the MCA, and the amount of variance accounted for by each dimension is shown in Table 4. Similarly to the approach adopted by Collett *et al*.^27^, only dimensions with a variance of 8% or more were retained for further analysis. This meant that Dimensions 1-4 (accounting for 37% of total variance) were retained for the CI group, and Dimensions 1-5 (accounting for 49% of total variance) for the NH group. Variance and eigenvalues for all dimensions were very similar across the two groups, suggesting similarity in the overall categorization strategy. The Supplementary Figure provides MCA maps for 10 dimensions per group, which accounted for 77% of variability in the CI group and 84% of variability in the NH group.

**Table 4:** Variance accounted for by each dimension of the MCA analysis and eigenvalues for both CI and NH children.

Dimension	Variance (%)		Eigenvalues	
	CI	NH	CI	NH
D1	10.12	11.51	0.84	0.90
D2	9.72	10.46	0.81	0.81
D3	8.93	9.84	0.74	0.77
D4	8.44	8.95	0.70	0.70
D5	7.35	8.14	0.61	0.63
D6	7.14	7.84	0.59	0.61
D7	6.62	7.60	0.55	0.59
D8	6.43	7.38	0.53	0.58
D9	6.13	6.34	0.51	0.49
D10	5.75	5.76	0.48	0.45
unaccounted	23.37	16.18		

A total of 10 dimensions were used in the analysis with the 4 most important retained.

**Figure 3:**
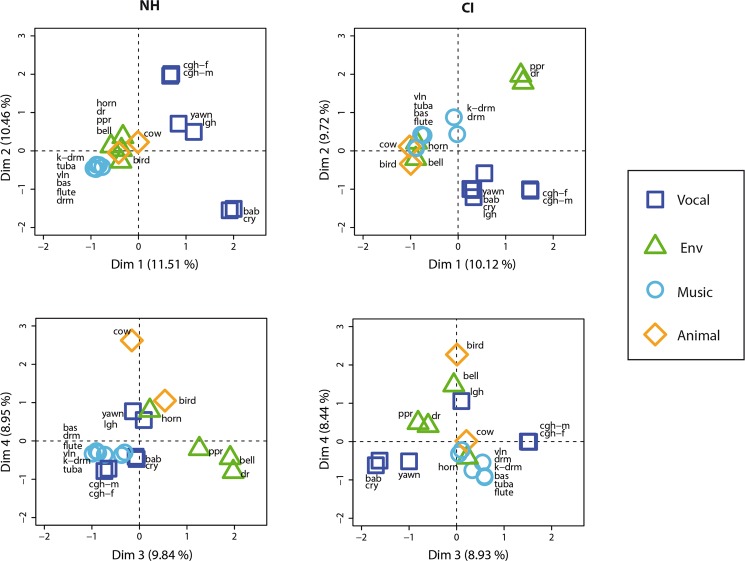
Factor maps of categories created by participants (sound stimuli). **S**timuli are plotted along the first four dimensions yielded by the MCA analysis (Dimensions 1 & 2 along the top row, and Dimensions 3 & 4 along the bottom row) for NH children (left column) and CI users (right column). The amount of variance accounted for by each dimension is given as a percentage on the x- and y-axes, and all plots use the same scale. Stimuli that lie on the dotted line (zero) are not considered to be part of the corresponding dimension. Finally, the stimuli are coloured according to the a priori categories and labelled using the abbreviated sound IDs given in Table 2.

Figure 3 represents the distributions of the stimuli across the first four dimensions for each participant group. For the NH group, Dimension (1 (top left panel) shows a clear distinction between vocalizations (specifically *cry* and *baby*) and musical sounds. Dimension 2 illustrates the distinction between different types of human vocalizations, specifically between baby and adult vocalizations. Dimension 2 also shows the pairing of vocal sounds (coughing sounds, infant sounds and *yawn* and *laugh* stimuli), as shown in the dendrogram (Fig. 2). The concentration of environmental sounds near the zero line of Dimensions 1 and 2 means that they were not strongly involved in either dimension. On Dimension 3, *door* and *bell* are paired together, and the proximity of the *paper* sound suggests that Dimension 3 separated these three sounds from the vocalizations and musical sounds. Finally, Dimension 4 shows the separation of a single sound (*cow*). The remaining dimensions are provided in the Supplementary Fig. 1, as explained variance was far lower and there was no practical benefit in interpreting them (e.g., Dimension 5 separates *paper* from *laughing*, Dimension 6 *laughing* from *cow*, and Dimension 7 *horn* from the others). These strategies separating a single sound from the others, but with reduced eigenvalues (Table 4), were only exhibited by a minority of participants.

The factor map for the implanted children had several similarities with the map for the NH children, especially between Dimension 2 for the CI group and Dimension 1 for the NH group (Panels a and b of Fig. 3), as confirmed by the significant Spearman correlation (rho = −0.7, *p* < 0.05). Both dimensions made a clear distinction between the human voice stimuli and the other sounds. Coughing sounds were paired in both groups. However, whereas there were clear pairs of vocal sounds for the NH group, this pattern does not occur for the CI group. Instead, there was a distinction between the coughing sounds and other vocalizations in Dimension 3.

### Participant maps

**Figure 4:**
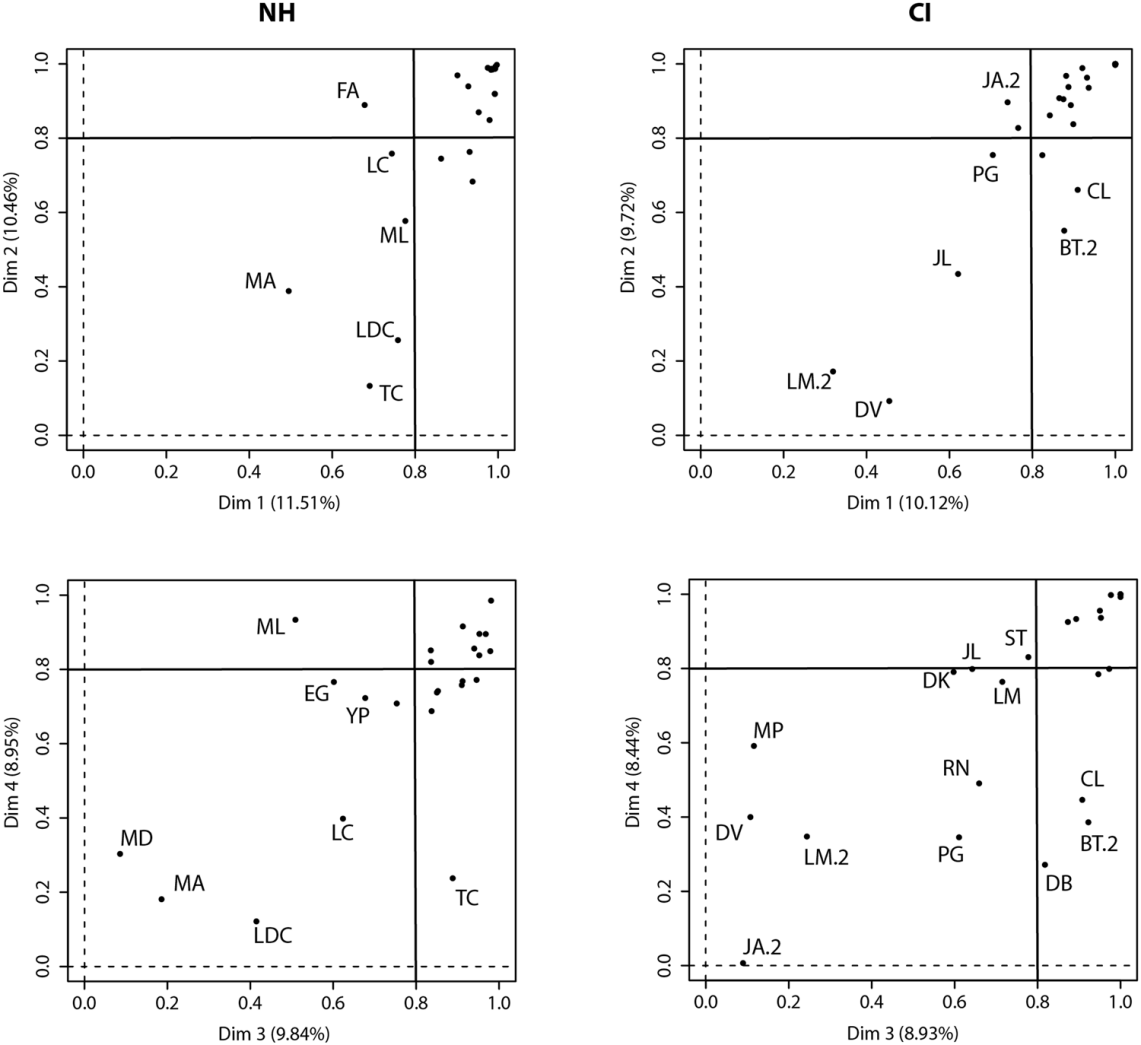
Factor maps of category variables (participants). Similar to Fig. 3, participants are plotted along the first two dimensions of the MCA analysis. This is done to show how strongly each participant adhered to the use of a particular dimension, with high values indicating strong adherence. To avoid clutter, subject codes are provided only for outliers (often below the contribution value of 0.8 for any dimension). Percentages are again given to show the variance covered by each dimension.

The distribution of participants along the four MCA dimensions is shown in Fig. 4, and can be interpreted to show how far participants used each of the dimensions in their categorization choices. NH and implanted children were similarly distributed across all four dimensions, and further similarities can be seen in the percentages of participants with coordinates greater than 0.8, as well as in the mean coordinate values (eigenvalues; see Table 4).

The distribution of participants across Dimensions 1 and 2 (top panels) shows that the majority of both NH and CI children had a coordinate value above 0.8 (NH: Dimension 1 = 75%, Dimension 2 = 67%; CI: Dimension 1 = 75%, Dimension 2 = 71%), indicating extensive use of these dimensions and their corresponding categorization strategies. There was a similar pattern of distribution for Dimensions 3 and 4 (lower panels), and the eigenvalues (Table 4) for these two dimensions were not much lower than those for Dimensions 1 and 2. Therefore, whilst fewer participants may have used Dimensions 3 and 4, the latter were nonetheless important to the children’s overall categorization strategy.

### Correlations with acoustic measures, participants’ data and identification performance

To achieve an objective interpretation of the dimensions yielded by the MCA, we analysed correlations with the acoustic characteristics of the sounds (see “Acoustic analysis” subsection for details). There was a significant link between certain acoustic characteristics and the categorization strategies of the children with CIs. For example, Dimension 3 correlated with max frequency (*r = *−0.63, *p* = 0.01) and cross-channel correlation values (*r* = 0.49, *p* = 0.04). Correlations are reported without correction for multiple comparisons.

Dimension 3 concerned the separation/grouping of the *baby*, *cry* and *yawn* sounds, which also had three of the five highest max frequency values (889, 842 and 890 Hz). The acoustic content of these three vocal sounds may therefore have been used to group them together. The correlation between Dimension 3 and cross-channel correlation was quite weak (*r* = 0.49), and was probably due to the high cross-channel correlation values for these sounds.

The correlation analysis in the implanted children also revealed a link between Dimension 1 and duration ratio (*r* = 0.49, *p* = 0.01). This was probably driven by the coughing sounds, as these were two of only five sounds with a value greater than zero. We also observed a correlation between Dimension 1 and fundamental pitch salience (*r* = −0.72, *p* < 0.001), probably resulting from the contrasting values of two groups of sounds. The *rustling paper* and *door* sounds had very low values (0.11 and 0.27), compared with the vocalized sounds, which ranged from 0.4 (*man coughing*) to 0.8 (*baby sound*).

After applying Holm’s correction for multiple comparisons, we found no significant correlations due to the large number of acoustic parameters. We decided to restrict the number of correlations to be similar to that previously reported in adult NH and CI listeners using the same paradigm^27^. In this case, we found 6 significant correlations for children with CIs and 3 significant correlations for normal-hearing children for dimensions 3 and 4. This suggests that there is a bias towards acoustically based categorization in children with CIs.

As frequency and temporal sound measures were inevitably more or less intercorrelated, we performed a principal components analysis (PCA). We found that the first component explained about 32% of variance and the second about 18%, making it impossible to reduce the data to principal components. However, this analysis did give us a general idea of how the two groups used acoustic parameters to categorize the sounds. This analysis confirmed that the children with CIs were more influenced by acoustic parameters than NH children were, especially for the first dimension of the MCA maps (denoted as DimCat 1 in Supplementary Fig. 2).

The implanted children’s coordinates on Dimensions 1–4 (see Fig. 4) were correlated with their demographic data (see Table 1). Results showed significant correlations between Dimensions 1 and 3 and age at implantation (*r* = 0.41 and 0.46, *p* < 0.05, not corrected for multiple comparisons), suggesting that these two dimensions may be more relevant for participants implanted at an older age. None of the other demographic data were correlated with the dimensions we retained.

There were no significant correlations between implanted children’s performances and their coordinates (Fig. 4), suggesting that the results of the MCA analysis were not linked to identification performance.

#### Overall performance on the free-sorting task

On average, the CI group took longer to complete the FST (mean ± *SD* = 724 s ± 296) than the NH group (566 s ± 202), although this difference was not significant (Kruskal-Wallis test, *p* = 0.06). There was also no significant difference between the groups on the mean number of categories created (8.8 ± 2 vs. 9.3 ± 1.5; Kruskal-Wallis, *p* > 0.05). Supplementary Table 2 shows the raw data and how the sounds were categorized by each participant. It illustrates the variability in participants’ categorization performances, with some children (both CI and NH) categorizing all 18 sounds separately, and others using a very limited number of categories - as low as three in some cases. Regarding listening strategy, we did not observe any significant difference between groups on the mean number of playbacks (NH: 5.45 vs. CI: 4.8).

**Figure 5:**
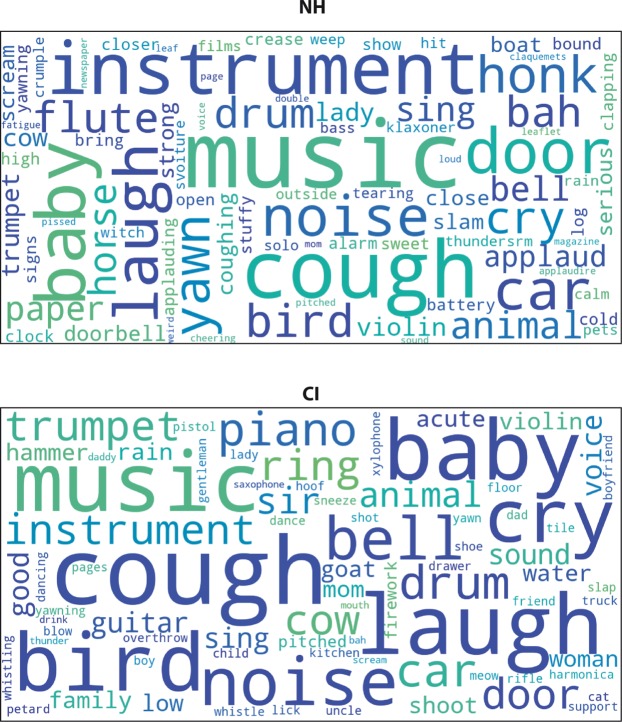
Word clouds of category descriptors. Word clouds show the words that were most used by participants to describe the categories they created in the FST (using Python package Wordcloud). The size the words reflects the frequency with which they were used by both groups (NH and CI). It should be noted that this figure does not reflect differences in the frequency of word usage between the groups (children with CIs generally used the words less frequently). For example, the word most frequently used by the children with CIs was *cough*, but they used it 30% less frequently than the NH children.

A word-cloud analysis was also conducted to visually display the verbal descriptors used by participants (Fig. 5). The size of each word reflects the frequency of usage (correct/incorrect) during testing and provides additional information to the identification performance of CI and NH groups. One can see that key words were similar for both participant groups, however they were less frequently stated by CI than NH. For example, “cough” was the word most frequently used word by CI participants, but was used 30% less than by NH participants.

To quantify the differences in vocabulary, we analysed the frequencies of words in stimuli descriptions used by the children with CIs and controls. Globally, children with CIs and controls used a similar number of words (79 and 83 correspondingly). Among the words used by NH children, 36% were also used by children with CI; most of these words corresponded to the presented stimuli or their categories (bird, door, cough, bell, animal, etc.). Thus, CI and NH children only had 36% of words in common during this task. We compared the frequency of usage of these “common” words between children with CIs and controls and found that the average frequency of word usage was 25% lower in children with CIs than in NH counterparts (p < 0.05, bootstrap). These results demonstrate that there is a considerable difference between children with CIs and NH children in the use of the vocabulary.

Overall, this analysis showed that the NH children and children with CIs performed the auditory FST in a similar manner.

## Sound-identification performance for children with CIs

Although verbal sound identification is not a reliable measure in children because of their poor vocabulary, we tried to analyse the identification of sounds by the children with CIs. A previous analysis in NH children revealed that they were able to use semantic-based categorization as early as 6 years of age^28^. In the present study, the NH children were not asked to identify any sounds, as identification performance lies outside the scope of the article. However, in the current study, we performed a supplementary identification analysis for the children with CIs to ensure that their performance is not random. On average, the implanted children correctly identified 35.3% of the 18 sounds (Response category 1): 30.8% in a stable manner (Response category 1A) and 4.5% in an unstable one (Response category 1B). These results show that children who are deaf with CIs have considerable difficulty identifying everyday sounds. While not precisely quantified, our previous study showed that age-matched NH children were able to identify all the sounds except for *rustling paper*^28^.

We also carried out a descriptive analysis of individual sounds. This showed that three of the 18 sounds were correctly identified more than 90% of the time (*women coughing*, *women laughing*, and *bird*). An additional sound (*baby crying*) was included when we also considered sounds that were partially identified. Most other sounds were poorly identified (see Fig. 1), especially the musical sounds.

We also ran a word-cloud analysis to visually display the verbal descriptors used by participants (Fig. 5). The size of each word reflects the frequency of usage (correct/incorrect) during testing, and provides additional information about the identification performances of the CI and NH groups. The word cloud shows that keywords were similar for both groups, although they were less frequently provided by the children with CIs than by the NH children. For example, *cough* was the word most frequently used by the former, but they nonetheless used it 30% less than the latter did. To quantify the differences in vocabulary, we analyzed the frequencies of words in stimuli descriptions used by the children with CI and controls. Globally, children with CI and controls used a similar number of words (79 and 83 correspondingly). Among the words used by NH children 36% were also used by children with CI, most of these words corresponded to the presented stimuli or their categories (bird, door, cough, bell, animal etc.). Thus, CI and NH children only have 36% of words in common during this task. We compared the frequency of usage of these “common” words between children with CI and controls and found that the average frequency of word usage was 25% lower in children with CI than in NH counterparts (p < 0.05, bootstrap). These results demonstrate that there is a considerable difference between children with CI and NH children in the use of the vocabulary. Besides, for the words in common between the two groups, which describe the more precisely the sounds, the frequency of usage is smaller in children with CI.

Overall, children with CIs rather accurately identified nonlinguistic human vocalizations and animal sounds, but had difficulty identifying musical and environmental sounds.

## Discussion

In the current study, we analysed and compared two groups of participants (NH children and children with CIs), who performed an FST featuring everyday sounds. These sounds included nonlinguistic human vocalizations, musical sounds, environmental sounds, and animal vocalizations. Results indicated that the two groups tended to perform the task in a similar way creating similar categories.

However, on closer inspection of the types of categories they created, coupled with a search for correlations with acoustic cues, we found that the children with CIs tended to adopt different strategies based more on a musical listening mode^24^, probably owing to deficits in sound identification abilities.

In our previous studies of sound categorization in adult CI users, we showed that, despite the deficit in voice discrimination^36,37^, experienced CI users are able to discriminate vocal sounds from broadly defined environmental and musical sounds^27^. We further showed that the categorization of vocal sounds with respect to non-vocal sounds improves during the post-implantation rehabilitation phase^38^. The first 6 months post-implantation appear to be crucial in this process. Our results in adults with CIs demonstrated that patients in different phases of rehabilitation use different acoustic cues, which increase in complexity with CI experience. In our study of NH children of different ages^28^, we showed an effect of age on auditory categorization, with 6 years appearing to constitute a pivotal stage, reflecting the gradual move away from non-logical reasoning based mainly on perceptual representations to the logical reasoning used by older children.

As previously mentioned, the NH children and children with CIs performed the auditory FST in a similar manner in the present study. The auditory FST not only elicits perceptual processes and cognitive representations, but also requires access to working memory, in order to compare and find similarities between the different sounds. The absence of significant differences in listening strategies between the two groups suggests that the children who are deaf with CIs did not have any deficits in the cognitive processes associated with the FST. Unlike the NH children, who treated the task as a game, most of the children with CIs exhibited attention deficits during the task. This observation requires further investigation of possible deficits in the higher cognitive functions of children with CIs that could result from developmental auditory deprivation, as postulated by the disconnection hypothesis^39^.

As explained in the Materials and Methods section, children with CIs were asked to name the stimuli in an open-set format, in order to verify their ability to identify everyday sounds. Although this task was not precisely quantified in the original study^28^, it did show that age-matched NH children were able to identify all the sounds we used in the current study. One notable exception was *paper rustling*, which was often confused with *applause*, *flapping wings* or *walking on fallen leaves*^28^.

In the current study, however, children with CIs had considerable difficulty identifying the 18 everyday sounds. Previous studies on children with CIs had already shown that they perform poorly on environmental sound recognition, compared with their hearing peers^16,17^. Using a closed-set format, they found that mean identification accuracy across all participants ranged between 59 and 68% for common environmental sounds. Our results showed a poorer performance for music sounds (35% correctly identified) because of the open-set response format^11,12,15^. When we included partial identification scores, corresponding to sounds identified either by a sound belonging to the same semantic family or by their superordinate category, we arrived at an overall identification accuracy of 58%, which is closer to that observed in previous studies.

Children’s experience hearing with their implant has also been shown to play an important role in their rehabilitation^16,17^. These authors found that the identification of environmental sounds was positively correlated with the duration of implantation, such that more hearing experience gave rise to higher identification scores.

In the current study, the duration of implantation was also positively correlated with identification performance. In addition, we found correlations between the MCA dimensions and both age at implantation and identification performance. This indirectly confirms that hearing experience could be used as a predictive factor of sound discrimination when undertaking the FST.

When we examined performances for the different a priori sound categories, our results revealed a major difference in identification between the vocalizations and other types of sounds. Nonlinguistic human vocalizations and animal vocalizations were identified with an accuracy of 80% or more (correct and partial identification taken together). By contrast, musical sounds and environmental sounds were poorly identified. At first glance, these results would appear to contradict previous evidence that implanted adults have great difficulty distinguishing the human voice from environmental sounds, as well as discerning attributes of the human voice such as age or gender^8,36,37,40–43^, resulting from the poor spectral information provided by the implant^44^. This apparent discrepancy between the current study and previous ones may stem from differences in the testing procedure. For example, when a two-alternative forced-choice task with short stimulus presentation is used, implanted adults cannot distinguish the human voice from environmental sounds^36^. By contrast, the current study used an FST, which allowed participants to listen to the stimuli multiple times, and a similar study demonstrated that implanted adults are especially efficient at grouping vocal sounds together^45^. Overall, in spite of the testing procedure, these results reinforce the well-known status of vocal sounds^46^, which are crucial for social communication, especially during early developmental stages^47^.

An overview of the final categorization choices showed that NH children adopted a categorization strategy whereby they essentially created many small groups of two or three sounds. This was probably due to semantic and acoustic similarities, and resulted in groups/pairs of baby voices, coughing, bass instruments (*tuba* and *double bass*) and percussion instruments (*kettledrum* and *drumkit*).

We had previously suggested^28^ that this pair-making strategy is a result of schooling. In France, children learn to develop analytical abilities using analogical comparisons and two-by-two associations. These are used for learning to read, based on the association between a phoneme and its written form. However, other categories are built up as stories or scripts^48^, based on the children’s own experience. This strategy of creating a story to categorize sounds was well represented in the 6-year-old group in our previous study^28^.

Grouping the sounds in pairs was not as common among the children with CIs. Results instead showed that they generally created one large category containing the musical sounds plus *cow* and *horn*, and four groups of two or three items. Their problems with sound identification may explain the larger group of musical sounds and the reduced discrimination of vocal sounds.

*Cow* and *horn* sounds were probably grouped with musical sounds because they were poorly recognized, thus making it difficult to categorize them based on their semantic cues. By contrast, they shared similar acoustic cues, notably pitch, with the musical sounds consisting of a single pitched note (*flute* and *violin*). Like the NH children, the children with CIs also created separate pairs of coughing and baby sounds, following an adjacency principle. These sounds share a common source, the human vocal tract, and therefore share many semantic and acoustic properties (e.g., mean frequency, spectral uniformity). Overall, the clustering analysis revealed clear similarities between the NH and CI groups. More specifically, CI and NH participants tended to group musical sounds together and human voices together - a strategy shared by CI and NH adults^27^.

MCA allows each sound to be represented as a data point in an *n*-dimensional Euclidean space based on the category choices made by participants. We used it to establish the influence of acoustically specific strategies and participants’ individual characteristics on categorization strategies.

The factor maps showed considerable intragroup heterogeneity for the categories created by both the NH children and the children with CIs. This was especially pronounced for Dimensions 3 and 4, for which showed no clear categorization strategy for either group.

Both groups exhibited a shared categorization strategy when discriminating between vocal and nonvocal sounds. This was observed for children with CIs along Dimension 2, and for NH children along Dimension 1. However, despite the similarity, it is unlikely that the two groups used the same strategy. For instance, correlation analysis (categorization dimensions vs. acoustic properties) applied to the NH children yielded less significant results, suggesting that NH children did not primarily use acoustic features for their similarity judgments. These results are in line with adult studies of auditory FST, showing that sound categorization relies on sound identification, and categories are primarily based on semantic information about sound‐producing objects/actions^24,25,49^. The acoustic characteristics of sounds, which determine listeners’ qualitative perceptions, play a less decisive role in auditory categorization, except when no semantic information is available^50^. This situation applies to children with CIs, who receive impoverished auditory spectral information, leading to much lower identification performances. Indeed, for the children with CIs in the present study, we found correlations between Dimensions 2 and 4 and the identification scores for individual sounds.

The results of the acoustic correlation analysis and PCA demonstrated that the implanted children used the acoustic properties of the sounds in their categorization strategies. These properties included max frequency (periodicity pitch measure), which was probably used by the children with CIs to group together the vocal sounds *baby*, *cry* and *yawn*. In addition, we found that cross-channel correlation was correlated with Dimension 3, and duration ratio with Dimension 1. A large body of research has shown that acoustic characteristics such as the spectrotemporal structure of sounds^11^, temporal envelope^13^, repetitive temporal structures^12^ or distinct temporal patterning^16^, and harmonic characteristics^12^ are extensively involved in sound identification in implanted individuals. Taken together, these results suggest that sounds were categorized and grouped together using multiple strategies combining semantic (source) information and the acoustic characteristics of the sounds. This means that the children with CIs had already acquired a strong capacity for the perceptual acquisition and semantic representation of vocal sounds, despite reduced auditory input. This is in line with previous results obtained with NH adults who performed a similar FST with vocoded sounds (eight and 16 channels)^45^. These results showed underlying similarities for the categorization of both natural and vocoded sounds. However, more varied categorization strategies and less distinct category groups were observed when the number of vocoder channels was reduced. Similar differences in performance were observed for implanted adults, though not as great. It therefore appears that, even with limited spectrotemporal information, NH adults perform better than CI adults. One might expect to observe a similar effect for children, owing to the impact of deafness and the possible disruption of the functional integrity of the auditory system at all levels. Studying children’s perception of vocoded sounds is a possible avenue for future research. However, it is one that would require careful experimental design controlling for task comprehension.

The analysis of correlations with the implanted children’s aetiological data revealed that Dimensions 1-3 may be related to age at implantation. This correlation, though uncorrected for multiple comparisons, underlines the notion that the earlier children obtain an auditory prosthesis, the better their abilities to process and categorize natural environmental sounds will be. While the mechanisms are different, these results can be seen in relation to data obtained in adult CI patients^38^, suggesting that FST performance is related to adaptation to the implant. We observed that the duration of CI experience influenced adults’ auditory categorization of a similar set of natural sounds. In particular, the voice/non-voice segregation was noticeably clearer after 6–9 months of experience with an implant.

Our data on children with CIs are supported by a vast body of literature demonstrating that early implantation is associated with optimum speech perception performance and facilitates language development^51–55^. The positive impact of implantation between 12 and 24 months has long been acknowledged, with numerous studies showing that the perceptual and language development of children implanted before 24 months is better than that of children implanted between 24 and 36 months^56–60^.

## Conclusions

The present study showed that children fitted with CIs are able to categorize everyday sounds in a similar way to typically developing NH children. In both groups, human vocal sounds - which are crucial for social communication - were clearly distinguished from other categories, despite the absence of speech content. Nonetheless, the implanted children seemed to use the *musical listening mode* more, and based their categorization choices on acoustic rather than semantic information. This approach is likely to be the result of poor identification abilities^61^. Further analysis highlighted the specific spectral and temporal acoustic variables that the children with CIs used to categorize everyday sounds. This could be important for future research into CI coding strategies and rehabilitation methods to reduce the deficits that CI users encounter in everyday auditory environments.

## Supplementary information

Supplementary information

## Data Availability

The main data analysed during this study are included in this published article and its Supplementary Information files. Other data are available from the corresponding author on reasonable request.
