# Supplementary material for: Categorization of everyday sounds by cochlear implanted children
Source: Sci Rep. 2019 Mar 5;9:3532. doi: 10.1038/s41598-019-39991-9 (PMC6401047; doi:10.1038/s41598-019-39991-9)
Supplement: Supplementary file 1 — Supplementary information [file 41598_2019_39991_MOESM1_ESM.pdf]

Categorization of everyday sounds by cochlear implanted children

Aurore Berland, Edward Collett , Pascal Gaillard, Michèle Guidetti, Kuzma Strelnikov,

Nadine Cochard, Pascal Barone, Olivier Deguine

Supplementary Figure 1. MCA maps for the higher dimensions

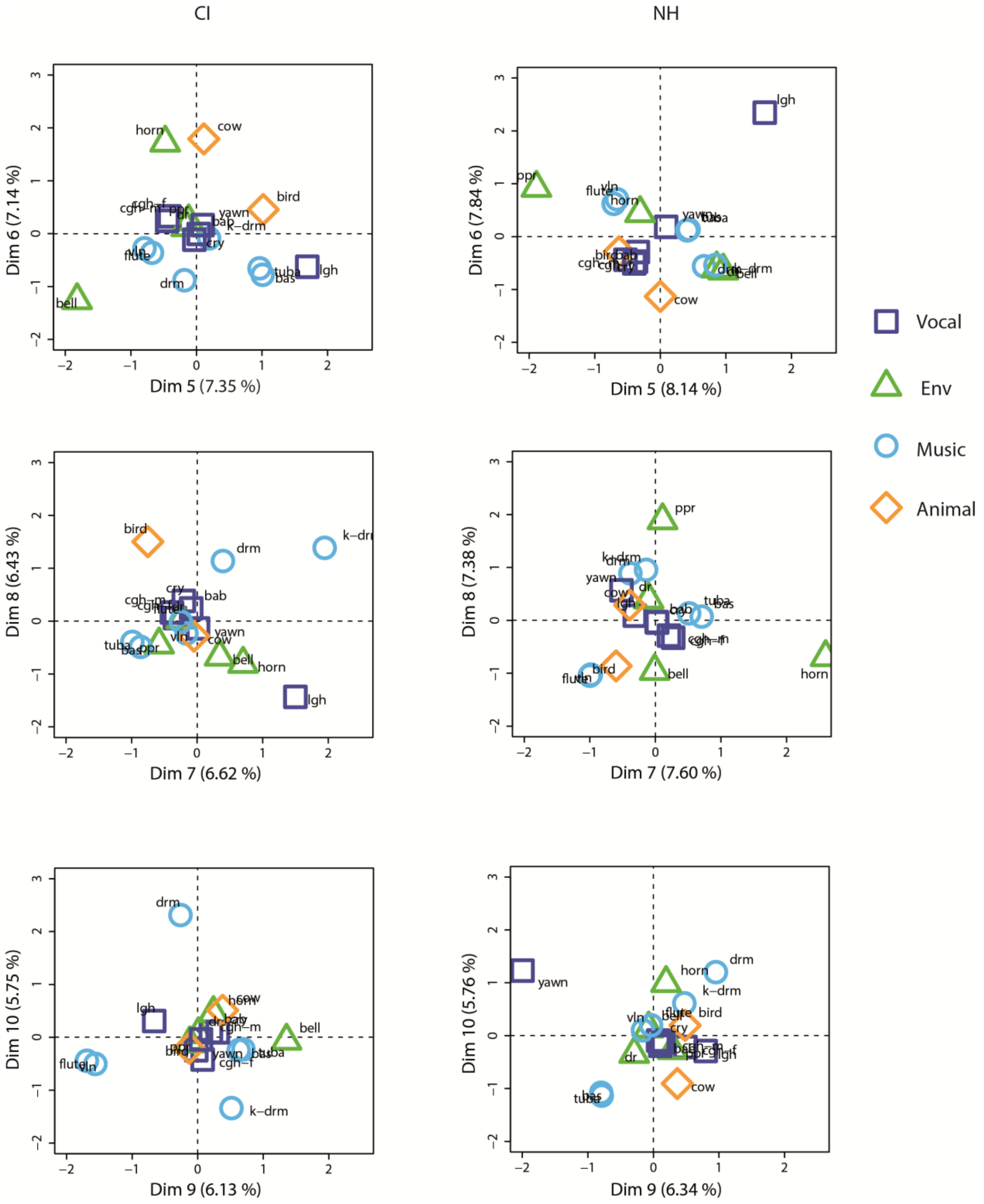

Supplementary Figure 2. PCA analysis of sound acoustic properties and the first two MCA dimensions (DimCat1 and DimCat2)

NH

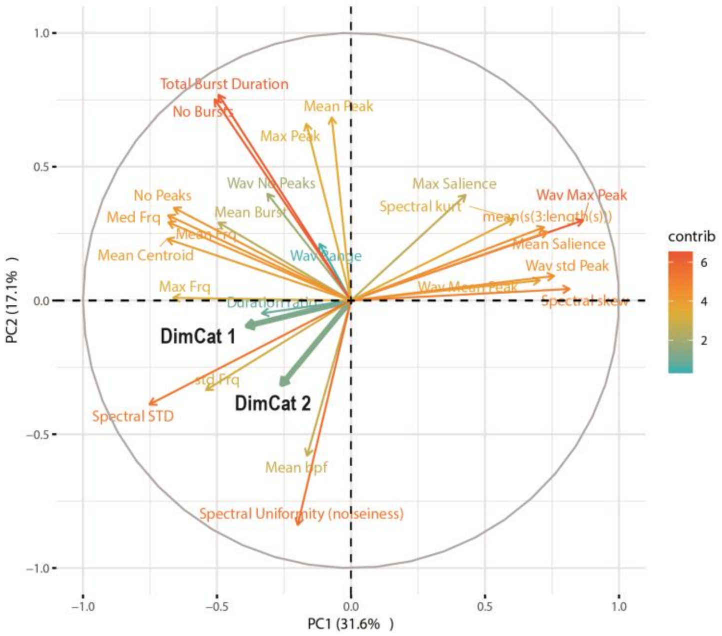

CI

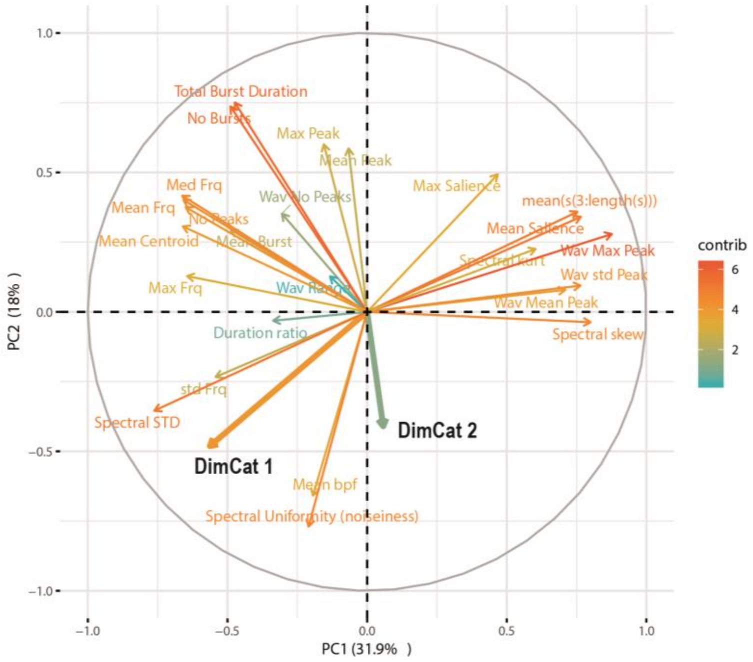

Supplementary table 1. Acoustic characteristics of sounds

Sound	Wav No Peaks	Wav Max Peak	Wav Mean Peak	Wav std Peak	Wav Range	Mean bpf
Rustling of paper	1.0	0.0	0.0	0.0	1.0	0.8
Front door	1.0	0.3	0.0	0.0	1.0	0.8
Drum kit	4.0	0.4	0.0	0.0	0.9	0.9
Cough man	1.0	0.6	0.0	0.0	0.9	0.8
Baby cry	1.0	0.4	0.0	0.0	0.9	0.6
Kettledrum	1.0	0.9	0.0	0.1	0.9	0.7
Bird	3.0	0.5	0.0	0.0	1.0	0.4
Cough woman	1.0	0.2	0.0	0.0	0.9	0.7
Laugh woman	5.0	0.2	0.0	0.0	1.0	0.6
Car horn	1.0	0.7	0.0	0.1	0.7	0.8
Yawn man	1.0	0.4	0.0	0.0	0.7	0.7
Cow	1.0	0.5	0.0	0.0	0.9	0.9
Babbling	1.0	0.7	0.0	0.0	0.9	0.6
Violin	1.0	0.7	0.0	0.0	0.9	0.4
Traverse flute	1.0	0.8	0.0	0.1	1.0	0.5
Double bass	0.0	0.9	0.1	0.1	0.9	0.6
Tuba	3.0	1.0	0.1	0.1	0.9	0.6
Door bell	0.0	0.9	0.2	0.3	1.0	0.6

Sound	No Bursts	Mean Burst	Total Burst Duration	Duration ratio	Max Peak	Mean Peak	No Peak
Rustling of paper	0.0	0.0	0.0	0.0	1371.5	873.6	5.0
Front door	0.0	0.0	0.0	0.0	2453.1	1543.0	3.0
Drum kit	5.0	40290.8	13903.0	6.3	6101.0	5836.1	5.0
Cough man	1.0	50727.0	2841.0	31.1	2372.4	2010.6	4.0
Baby cry	1.0	81402.0	2859.0	30.9	2244.7	1823.2	2.0
Kettledrum	0.0	0.0	0.0	0.0	3485.8	3485.8	1.0
Bird	9.0	43654.8	26538.0	3.3	6230.4	4304.6	8.0
Cough woman	3.0	38467.3	6176.0	14.3	2832.3	1984.4	6.0

**Laugh woman**	0.0	0.0	0.0	0.0	2293.3	1288.2	7.0
**Car horn**	0.0	0.0	0.0	0.0	2733.5	2037.4	2.0
**Yawn man**	0.0	0.0	0.0	0.0	2327.4	1876.4	2.0
**Cow**	0.0	0.0	0.0	0.0	2196.3	1755.4	3.0
**Babbling**	0.0	0.0	0.0	0.0	9529.8	5400.3	3.0
**Violin**	0.0	0.0	0.0	0.0	1620.7	1148.0	3.0
**Traverse flute**	0.0	0.0	0.0	0.0	2285.5	1258.6	6.0
**Double bass**	0.0	0.0	0.0	0.0	1643.6	1256.7	2.0
**Tuba**	0.0	0.0	0.0	0.0	3377.9	3140.9	2.0
**Door bell**	0.0	0.0	0.0	0.0	1538.4	1538.4	1.0

Sound	Mean Frq	Med Frq	std Frq	Max Frq	Mean Salience	Max Salience
**Rustling of paper**	455.8	444.4	272.2	842.1	0.1	0.2
**Front door**	327.4	304.8	240.1	761.9	0.3	0.9
**Drum kit**	122.3	97.0	52.2	202.5	0.3	0.8
**Cough man**	148.2	160.0	64.8	290.9	0.4	0.6
**Baby cry**	475.0	470.6	142.3	842.1	0.4	0.8
**Kettledrum**	99.8	88.8	43.1	216.2	0.5	0.7
**Bird**	756.4	800.0	124.5	941.2	0.5	1.0
**Cough woman**	524.8	666.7	251.9	800.0	0.6	0.9
**Laugh woman**	402.9	326.7	180.1	842.1	0.6	0.8
**Car horn**	156.1	135.6	70.0	246.2	0.6	0.9
**Yawn man**	339.5	287.2	247.8	888.9	0.6	1.0
**Cow**	231.5	183.9	116.5	571.4	0.8	1.0
**Babbling**	401.2	410.3	132.6	888.9	0.8	1.0
**Violin**	661.6	666.7	276.1	941.2	0.9	1.0
**Traverse flute**	303.7	192.8	240.6	761.9	1.0	1.0
**Double bass**	89.3	89.4	0.2	89.4	1.0	1.0
**Tuba**	137.8	132.2	35.1	173.9	1.0	1.0
**Door bell**	224.2	175.8	96.4	410.3	1.0	1.0

Sound	Spectr. Uniformity (noiseiness)	Mean Centroid	Mean Frq	Spectral STD	Spectral skew	Spectral kurt	RMS	Spectr Velocity
**Rustling of paper**	0.7	2553.3	455.8	1265.0	0.0	-1.0	0.0	11969.5
**Front door**	0.7	1391.9	327.4	1236.7	1.1	0.4	0.1	6649.0
**Drum kit**	0.3	1212.4	122.3	1437.7	1.1	-0.2	0.2	5078.6
**Cough man**	0.5	1187.1	148.2	1070.8	1.2	1.0	0.0	5382.2
**Baby cry**	0.6	2163.0	475.0	1036.8	0.6	-0.4	0.0	9714.7
**Kettledrum**	0.3	628.8	99.8	721.1	2.4	6.6	0.0	2109.8
**Bird**	0.1	3761.2	756.4	836.0	-1.2	3.4	0.0	8051.7
**Cough woman**	0.6	1393.5	524.8	1240.0	1.2	0.5	0.0	7310.8
**Laugh woman**	0.7	1824.7	402.9	1194.3	0.7	-0.7	0.0	4570.2
**Car horn**	0.6	2118.9	156.1	1092.1	0.5	-0.7	0.0	5308.3
**Yawn man**	0.5	1091.4	339.5	1113.7	1.4	1.2	0.0	4211.5
**Cow**	0.8	2042.0	231.5	1112.8	0.4	-0.6	0.0	4737.8
**Babbling**	0.4	1407.4	401.2	1036.2	1.8	3.0	0.0	0.0
**Violin**	0.6	2206.5	661.6	1192.9	0.4	-0.9	0.0	6840.5
**Traverse flute**	0.4	1011.1	303.7	739.7	2.4	6.4	0.0	2690.5
**Double bass**	0.4	618.7	89.3	699.4	2.8	10.2	0.2	1532.8
**Tuba**	0.4	341.0	137.8	326.3	6.0	56.4	0.0	2451.0
**Door bell**	0.4	1676.1	224.2	767.7	1.9	3.5	0.0	3267.0

Supplementary table 2. Raw categorization data

NH children, raw categorization

All categories being numbered per subject, the value indicates into what category each sound is placed.

		lgh f	drum	bell	cgh f	door	drum	paper	babble	yawn	klaxon	bird	bass	baby	tuba	violin	cow	cgh m	flute		Nb cat
subject codes	AD	10	6	5	7	5	6	9	1	8	9	4	3	1	3	2	4	7	2		10
	CD	3	8	7	9	3	1	4	2	6	4	7	1	2	1	5	10	6	5		10
	CL	6	4	7	2	5	8	5	3	9	10	11	4	3	4	1	12	2	1		12
	EG	1	7	5	1	5	5	6	4	1	8	6	2	4	2	3	9	1	3		9
	EL	8	5	9	2	11	12	10	1	7	6	4	3	1	4	3	6	2	4		12
	FA	4	1	2	6	2	1	8	3	7	3	7	5	3	5	8	4	6	8		8
	JC	4	5	7	2	6	5	6	1	4	7	8	3	1	3	8	9	2	8		9
	LA	6	3	4	1	4	3	7	2	8	9	5	3	2	3	3	5	1	3		9
	LB	8	4	7	5	7	3	9	1	5	6	2	3	1	3	4	2	5	4		9
	LC	8	5	1	6	3	2	4	8	3	9	4	5	1	9	7	2	6	7		9
	LD	6	2	5	4	5	2	7	1	8	3	3	2	1	2	2	3	4	2		8
	LDC	1	4	2	4	3	4	5	1	1	4	2	4	1	4	2	4	3	2		5
	LL	5	3	8	1	2	3	2	4	7	5	8	6	4	6	9	7	1	10		10
	LR	1	2	4	3	4	2	7	1	1	6	5	2	1	2	2	8	3	2		8
	MA	1	3	4	2	2	5	5	1	5	5	4	5	3	5	4	5	5	4		5
	MD	1	3	5	1	6	6	4	2	4	5	5	5	2	6	5	3	4	5		6
	ML	3	1	3	1	7	7	4	5	9	8	8	2	5	2	4	10	6	6		10
	OL	9	6	5	1	10	6	7	3	1	5	4	2	3	2	11	8	1	4		11
	OS	3	2	1	5	1	6	8	4	3	9	2	6	4	6	7	10	5	7		10
	PA	8	7	9	2	5	7	5	1	6	11	10	3	1	3	4	6	2	4		11

	TB	9	7	2	1	2	7	5	3	10	8	6	8	3	4	4	6	1	5		10
	TC	2	1	3	2	4	1	6	2	2	5	2	1	2	1	1	2	2	1		6
	YP1	1	7	2	1	2	7	7	3	6	4	5	4	3	7	7	6	1	7		7
	YP2	7	2	1	4	1	2	1	3	3	5	1	5	3	5	5	6	4	5		7
																			**Mean**		8.79
																			**SD**		1.99
																			**bootstrap CI**		7.96<br>9.54

CI children, raw categorization

		lgh f	drum	bell	cgh f	door	drum	paper	babbling	yawn	klaxon	bird	bass	baby	tuba	violin	cow	cgh m	flute		Nb cat
subject codes	AC	1	2	3	4	5	6	7	8	16	9	10	11	12	13	14	15	17	18		18
	AM	4	7	5	13	6	9	8	3	2	1	4	2	3	2	10	1	12	11		13
	BT1	1	2	3	4	5	6	7	15	8	9	10	17	16	18	11	12	13	14		18
	BT2	8	1	2	5	4	7	4	3	3	7	9	8	4	6	2	6	5	9		9
	CA1	4	10	5	6	9	13	12	1	1	2	8	3	1	3	2	11	7	2		13
	CA2	12	3	7	4	3	10	11	2	9	6	8	5	2	5	1	6	4	1		12
	CL	3	1	1	2	3	1	3	4	4	1	5	1	4	1	1	5	2	1		5
	DB	3	1	1	6	2	8	2	5	5	7	4	4	5	4	5	4	6	1		8
	DK	2	1	3	5	6	1	6	2	2	4	3	1	1	1	1	4	5	1		6
	DV	1	3	1	1	1	3	1	1	1	1	2	3	1	3	3	2	1	3		3
	JA1	4	5	6	2	7	8	9	10	11	1	12	3	13	3	14	1	2	15		15
	JA2	1	2	2	1	3	3	3	1	1	2	2	2	1	2	2	2	1	2		3

	JL	4	1	5	3	3	1	1	2	2	1	4	1	2	1	1	1	3	1		5
	KR	7	9	8	3	4	4	10	2	1	1	11	6	2	6	5	1	3	5		11
	LJ	11	2	3	18	16	5	6	17	7	8	4	10	1	12	13	14	15	9		18
	LM1	8	6	3	8	4	8	4	1	8	7	3	7	1	2	5	2	6	5		8
	LM2	1	1	2	1	1	1	1	3	3	3	2	1	2	1	3	3	1	3		3
	MP	6	6	3	1	7	7	7	6	1	4	5	2	6	2	3	3	6	3		7
	MR	6	3	8	9	7	1	7	2	2	8	6	10	2	10	5	4	9	5		10
	MS	4	1	5	3	6	1	7	2	2	1	8	1	2	1	1	1	3	1		8
	PA	3	4	3	2	1	4	1	5	6	8	9	7	5	7	8	9	2	6		9
	PG	5	3	1	3	6	7	8	1	1	4	1	2	1	2	1	1	3	1		8
	RN	1	6	5	1	2	6	2	3	1	4	5	6	3	6	5	4	1	5		6
	ST	6	1	5	6	1	4	1	3	3	4	7	2	3	2	2	4	6	2		7
																			**Mean**		9.29
																			**SD**		4.62
																			**bootstrap CI**		7.63 11.29

Supplementary Table 3. Identification scores per response category according to chosen criteria (explained in the text)

	**Category 1**		**Category 2**		**Category 3**	
	A	B	C	D	E	F
Environm.	21.20%	2.90%	6.70%	0.00%	53.80%	15%
Music	3.80%	1.20%	22.40%	13.50%	46.20%	13%
Animal	63.50%	7.70%	7.70%	1.90%	17.30%	2%
Vocal	53.20%	7.70%	18.60%	7.00%	9.00%	5%
